# Rare but Foreseeable: Rapidly Expanding Retropharyngeal Hematoma After Fall from Height

**DOI:** 10.5811/cpcem.2021.2.51119

**Published:** 2021-03-12

**Authors:** Alexander Bracey, Christine S. Ahn, Ryan N. Barnicle, Michael P. Frost, Marshall N. Leonard, Brian J. Wright

**Affiliations:** * Albany Medical Center, Department of Emergency Medicine, Albany, New York; † Stony Brook University, Department of Emergency Medicine, Stony Brook, New York

**Keywords:** Critical care, airway, trauma, retropharyngeal hematoma

## Abstract

**Case Presentation:**

An elderly man presented to the emergency department after a fall from a 15-foot height. Initial examination revealed signs of head and neck trauma without airway compromise. Computed tomography imaging identified cervical fractures at the first and second level with a retropharyngeal hematoma. In discussion with the trauma service, the patient was admitted to the hospital for airway monitoring. After 10 hours he clinically deteriorated, resulting in acute respiratory failure, and ultimately required intubation. The patient was intubated with a hyperangulated video laryngoscopy, and a surgical set-up was also prepared. The intubation was uncomplicated and resulted in clinical improvement. The patient was extubated after three days without difficulty and was ultimately discharged following an uncomplicated hospital course.

**Discussion:**

Retropharyngeal hematoma is a rare but significant clinical condition. Rapid decline and airway compromise have been described. Patients often require intubation and mechanical ventilation to avoid airway obstruction and respiratory failure. Coagulopathies should be reversed, if present. Prompt recognition and treatment of this condition is crucial to successful management.

## CASE PRESENTATION

**Image 1:**
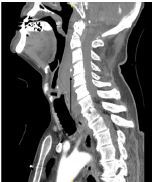
Computed tomography neck angiography demonstrating retropharyngeal hematoma with contrast extravasation (arrow) at the third and fourth cervical level, sagittal view.

**Image 2:**
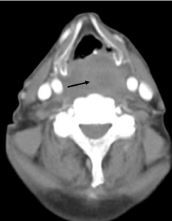
Computed tomography neck angiography demonstrating a 2.72-centimeter retropharyngeal hematoma, axial view (arrow).

A 79-year-old man with Parkinson dementia presented to the emergency department after an unwitnessed fall from a 15-foot ladder. The patient was amnestic to the event. He did not take anticoagulant or antiplatelet medications. He had facial bruising on exam and midline cervical spine tenderness, but no stridor or increased respiratory effort. The trauma team was activated, and the patient underwent emergent computed tomography, including arterial angiography of the head and neck (Image 1 and 2) as part of routine screening for blunt cerebrovascular injury. These images revealed displaced fractures at the first and second cervical levels with associated active retropharyngeal hematoma at the level of the hypoglottis.

Computed tomography of the chest, abdomen, and pelvis were also obtained as part of a trauma Level 1 activation order set. Nearly 10 hours after initial presentation, the patient developed respiratory distress and desaturated, requiring endotracheal intubation. This was performed by anesthesia using hyperangulated video laryngoscopy with an unobstructed view of the glottic structures resulting in a first-pass success and clinical improvement. Neurosurgery was consulted for possible embolization; however, no intervention was performed as it was a venous bleed. The patient was extubated after three days without complication and was discharged after an uncomplicated hospital course.

## DISCUSSION

Retropharyngeal hematoma is a rare but important clinical condition. The retropharyngeal space is small, with the average space between the vertebral body and the posterior aspect of the upper airway ranging between 3–14 millimeters depending on the cervical level.^1^ Blunt and penetrating neck trauma is a common cause, and it also appears to be associated with anticoagulant use and displaced cervical spine fracture.^2^,^3^ Other reported causes include vigorous coughing, retropharyngeal infection, local surgery, pharyngeal foreign bodies, spontaneous hemorrhage, and parathyroid tumors.^4^ Risk factors associated with the development of retropharyngeal hematoma include anticoagulant use, coagulopathies, vascular lesions, and vertebral bone disorders. Rapid decline can occur especially when signs of respiratory compromise exist, which may result in anoxic injury and death; therefore, emergency physicians should have a low threshold to secure the airway and coagulopathies should be promptly reversed if present.^3^,^5^

A double endotracheal and surgical airway setup should be strongly considered, as it may not be possible to pass an endotracheal tube from above the larynx depending on the size and extent of the hematoma.^4^ Emergent surgical airway (eg, cricothyroidotomy, tracheostomy) may be preferrable to endotracheal intubation in the presence of airway obstruction to avoid disruption of the hematoma.^6^ High suspicion and prompt management are necessary to successfully treat patients present with retropharyngeal hematoma. Definitive treatment may include arterial embolization or decompressive surgery, although conservative management is often adequate.^6^, ^7^

**CPC-EM Capsule**

What do we already know about this clinical entity?

*Retropharyngeal hematoma is rare. Displaced cervical fractures and anticoagulant use are risk factors for retropharyngeal hematoma and may cause airway compromise.*

What is the major impact of the image(s)?

*The images demonstrate that the retropharyngeal space is small. Even relatively minor bleeding in this space can compress airway structures and cause respiratory failure.*

How might this improve emergency medicine practice?

*This disease may go unrecognized initially as signs of airway compromise may be delayed. Standard and surgical airway set-ups should be prepared if the airway must be controlled.*
